# Computational Pathology Assessments of Cardiac Stromal Remodeling: Clinical Correlates and Prognostic Implications in Heart Transplantation

**DOI:** 10.21203/rs.3.rs-4364681/v1

**Published:** 2024-05-15

**Authors:** Eliot Peyster, Cai Yuan, Sara Arabyarmohammadi, Priti Lal, Michael Feldman, Pingfu Fu, Kenneth Margulies, Anant Madabhushi

**Affiliations:** University of Pennsylvania; Emory University; Emory University; University of Pennsylvania; Indiana University; Case Western Reserve University; University of Pennsylvania; Emory University

**Keywords:** Digital Pathology, Cardiac remodeling, Cardiac Stroma, Allograft Rejection, Heart Transplantation

## Abstract

Both overt and indolent inflammatory insults in heart transplantation can accelerate pathologic cardiac remodeling, but there are few tools for monitoring the speed and severity of remodeling over time. To address this need, we developed an automated computational pathology system to measure pathologic remodeling in transplant biopsy samples in a large, retrospective cohort of n=2167 digitized heart transplant biopsy slides. Biopsy images were analyzed to identify the pathologic stromal changes associated with future allograft loss or advanced allograft vasculopathy. Biopsy images were then analyzed to assess which historical allo-inflammatory events drive progression of these pathologic stromal changes over time in serial biopsy samples. The top-5 features of pathologic stromal remodeling most strongly associated with adverse outcomes were also strongly associated with histories of both overt and indolent inflammatory events. Our findings identify previously unappreciated subgroups of higher- and lower-risk transplant patients, and highlight the translational potential of digital pathology analysis.

## Introduction

Immune-mediated damage of the transplanted heart is the primary cause of allograft failure and a primary focus of post-transplant medical care.^1–4^ Established approaches for evaluating and characterizing the inflammatory processes affecting allograft health predominantly focus on the inflammatory cells themselves, rather than the long-term effects of inflammation.^1–4^ Endomyocardial biopsies (EMB) are routinely obtained, undergoing histologic grading based on the number, extent, and impact of infiltrating immune cells^1,5^. Newer ‘biopsy free’ serologic assays which are designed to reduce the burden of surveillance EMBs patients undergo also focus on measurements of immune cell activity, along with markers of active inflammatory damage, in order to assess allograft health.^6–9^

While infiltrating immune cells are a primary cause of allograft injury, these cells themselves may not reflect the persistent allograft injury that has occurred. The morphologic correlates of the sustained allograft dysfunction which may result from inflammatory insults are mainly stromal processes affecting the extracellular matrix (ECM),^10–13^ and are subtle and difficult to standardize with conventional histologic assessments. The cardiac stroma consists predominantly of fibroblasts, which undergo activation and differentiation under inflammatory conditions, leading to ECM remodeling via the deposition of thicker, more disordered collagen fibers.^14,15^ Remodeling of the cardiac stroma perturbs normal myocardial structure, increasing stiffness, transducing maladaptive signals, and hindering substrate delivery and metabolic waste removal.^10–13,15–18^ Indeed, it is long-term stromal remodeling driven by chronic and/or temporally remote immune-mediated damage that is thought to be the mechanism of ‘stiffening’ observed in failing cardiac allografts and in those affected by cardiac allograft vasculopathy (CAV).^12,16,18–20^

There are no existing tools for measuring the micro-architectural changes that result from inflammatory processes in cardiac allografts. We assert that this represents an important limitation, both in documenting the effects of acute immune-mediated insults such as severe rejection events, and in tracking the longer-term effects of more indolent, sub-clinical processes such as recurrent low-grade rejection or Quilty lesions. An assay capable of detecting the early, microscopic sequelae of pathologic remodeling could identify at-risk patients long before the development of overt, symptomatic, allograft dysfunction. This in turn could provide opportunities for early interventions, either in the form of intensified immunosuppression to quash indolent inflammation, therapeutics which have established roles in blunting pathologic remodeling in native hearts or emerging approaches that directly target stromal remodeling.^21^

In this study, we leverage computational pathology analysis to deeply interrogate the stromal microarchitecture of transplant EMB histology slides. Unlike recent studies utilizing computational pathology to assign International Society for Heart and Lung Transplantation (ISHLT) grades to EMB slides, the present study does not seek to reproduce work already performed by pathologists.^5,22^ Instead, the focus is on developing a morphologic assay capable of extracting previously unavailable information from EMB histology samples via a rigorous, quantitative, first-in-field analysis of allograft stroma. Deploying this novel stromal assessment tool in more than 2000 EMBs, we first establish the set of stromal ‘morphologic biomarkers’ most strongly associated with long-term adverse allograft outcomes. We then evaluate how these novel morphologic biomarkers change over time following various allo-immune insults, assessing the impact on stromal remodeling of both overt allograft injury in the form of treated high-grade rejection, and the impact of untreated, indolent inflammation in the form of recurrent, low-grade rejection and Quilty lesions. By quantifying the specific impact on a tissue- and patient-level of these historical insults, we provide new insights into allograft biology and challenge conventional wisdom on which allo-immune processes merit therapeutic intervention.

## Methods

### Study cohort description, image quality control and color normalization

**Figure 1:**
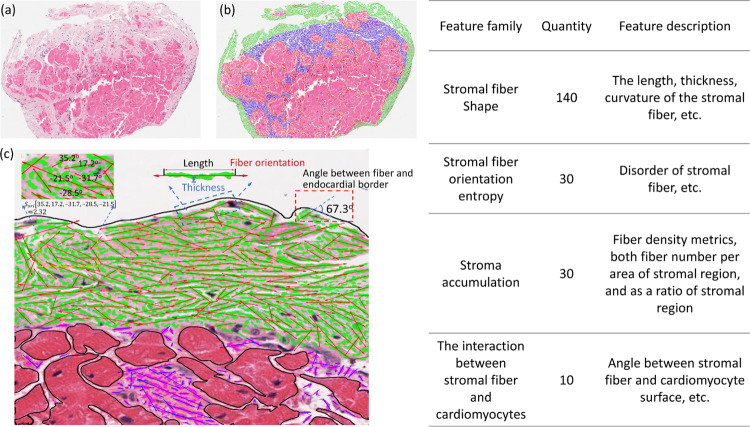
Study overview. At the top, a flowchart outlines the number of endomyocardial biopsy (EMB) slides in each inflammatory category. All EMBs can be categorized into one of six subgroups according to the historical events experienced by the patient prior to EMB acquisition, as shown in the figure. The middle section summarizes the computational pathology pipeline, including image quality control and color normalization, stromal morphologic feature segmentation and selection, and analysis of how each stromal feature changes over time between different inflammatory history subgroups. The length of the lines in middle section Panel 3 respectively represent the size and orientation of stromal fibers. Different colors represent different types of stromal fibers in panels 2 and 3 (green: endocardial stroma, red: interstitial stroma, blue: replacement stroma). The final section of the Figure shows the number of patient-level and EMB-level adverse outcomes experienced in the cohort, with adverse outcomes defined as the composite of allograft loss or CAV within 7-years.

The study cohort consisted of 2167 hematoxylin and eosin (H&E) stained EMB histology slides obtained from 650 patients treated between 1999 and 2016 at the Hospital of the University of Pennsylvania (UPenn). Study cases were derived from a combination of four previously published cohorts.^20,23–25^ Due to our interest in monitoring serial samples and progressive morphologic changes, EMBs from patients who had not received at least 3 EMBs post-transplant were excluded. Due to our interest in the impact of historical - rather than active – rejection events on allograft stroma, all study EMBs were confirmed to be free from serious rejection. Specifically, we excluded EMBs with any acute cellular rejections (ACR) ≥ grade 2R, any antibody-mediated rejections (AMR) with a pathological AMR grade ≥ grade 1,^1,4^ and all treated rejection regardless of grade. Histologic and clinical rejection diagnoses (cellular and antibody rejection grades, treated rejection history, and ‘Quilty’ lesion presence) for all preceding EMB events were compiled for each study EMB, with totals for each diagnosis aggregated. Study EMBs were then assigned to the following ‘inflammatory history’ subgroups: **1**) all patients with a previous high-grade rejection (≥ 2R, ≥pAMR-1), or a treated rejection not otherwise specified), **2**) the subset of the subgroup 1 patients without recurrent low-grade or Quilty, **3**) patients with recurrent low-grade rejection (≥ 3 grade 1R EMBs without any history of high-grade/treated rejection), **4**) the subset of the subgroup 3 patients with recurrent low-grade *and* recurrent Quilty lesions (with ≥ 3 occurrences of each diagnosis) and **5**) frequent inflammatory events (any patient with ≥ 5 prior histologic diagnoses of Quilty, ACR ≥ 1R, and/or AMR ≥ pAMR-1), and **6**) controls, who met none of the other group inclusion criteria. See Fig. 1 for additional cohort details. Clinical data was collected for all patients contributing an EMB to the cohort, including selected, established or purported donor and recipient risk factors for adverse allograft outcomes as listed in Table 1. Using these data, we identified patients with or without “adverse outcomes” defined as allograft loss (cardiovascular death or re-transplantation) or ≥ grade 2 CAV by 7-years post-transplant. This research complies with the Declaration of Helsinki, and access to archival data and tissue was approved by the University of Pennsylvania Institutional Review Board, with waiver of consent authorized by 45.CFR.46.116(d) and 45.CFR.164.512(i).

All slides were digitized via whole-slide scanning at 40x magnification. Digitized slides underwent quality control assessments using HistoQC, an open-source, quantitative digital pathology analysis software tool for identifying artifacts and measuring slide quality.^26^ A total of 265 slides were excluded from the study due to significant staining artifacts or damage to the archival slide. Color normalization was applied to each slide to account for slide and batch variations that can negatively impact image segmentation performance.^27^

### Stromal fiber detection and feature analysis

**Figure 2:**
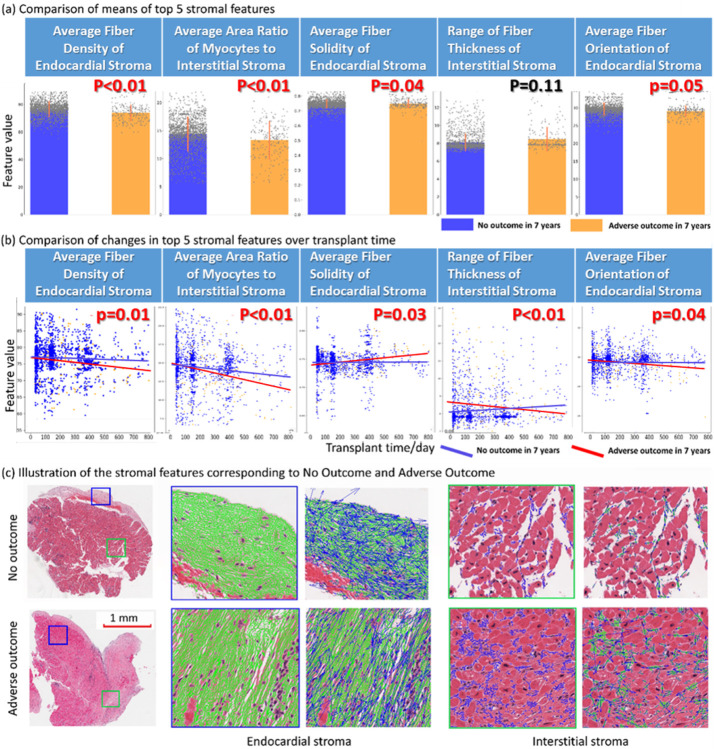
Stromal feature identification workflow and annotated examples. The image on the left shows the workflow of feature extraction, including the division of stroma types, stromal fiber segmentation, and the extraction of stromal features. (a) Original H&E tissue staining image. (b) The effect of stroma type division (different colors represent different stroma types, green: endocardial stroma, red: interstitial stroma, blue: replacement stroma). (c) Representative examples of several extracted stromal features. The yellow box illustrates the local orientation entropy with the proximal 5 fibers. The blue box shows the length, thickness, and orientation of the fiber. The red box indicates the angle between the endocardial fiber and the endocardium boundary. The right table displays a summary of feature categories examined including: fiber shape, orientation, stromal accumulation, and the interaction between fibers and myocytes.

Considering that different inflammatory processes act on different areas of cardiac stroma,^2,11,12,14,17,18^ three distinct stromal sub-regions were considered for subsequent morphologic feature extraction: 1) endocardial stroma, 2) interstitial stroma, and 3) replacement stroma (Fig. 2b). A total of 210 stromal features were extracted from each EMB slide. Broadly, these features pertain to four distinct categories, as outlined in Fig. 2, and described in more detail in Table 2 and Supplemental Methods. The first category describes the shape and size of stromal fibers: fiber length, fiber thickness (the total pixel size of the fiber divided by the fiber length), fiber solidity (the pixel ratio of a fiber and its smallest convex polygon), and fiber perimeter (obtained by the eight-direction chain code). The second feature category characterizes spatial orientation - the degree of local order/disorder - of stromal fibers, calculated locally within a 200-pixel neighborhood. The third category describes the accumulation and proliferation of stroma, including spatial density of the fibers (the number of stroma fibers in a certain pixel area) and the area ratio of cardiomyocytes to interstitial stroma. The fourth category describes the interaction between stromal fibers and cardiomyocytes, including assessing the angle and proximity of stromal fibers to the surrounding cardiomyocytes.

#### Segmentation method of stromal fibers:

**Figure 3:**
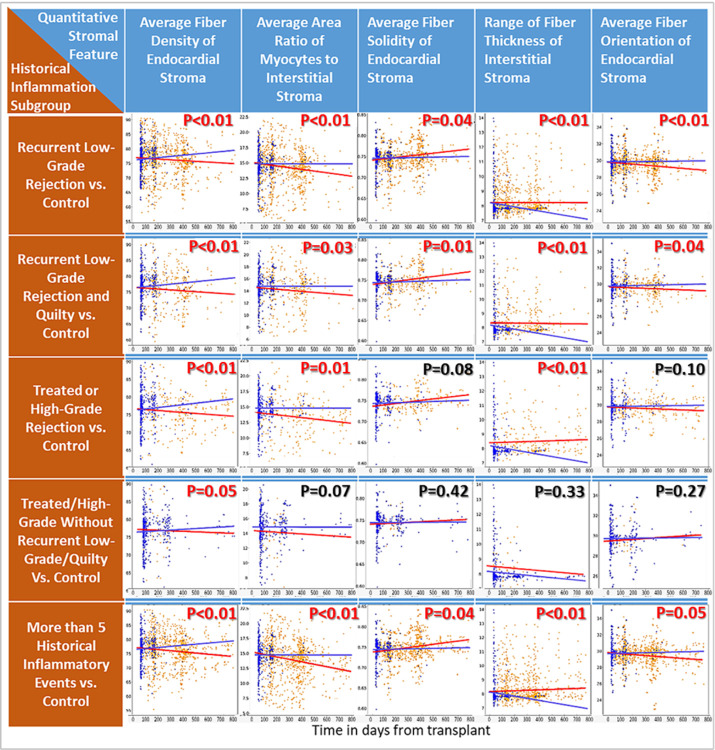
Prioritized stromal biomarkers of remodeling, identified based on significant changes over time from transplant between the Composite Adverse Outcome group and the No Outcome group. (a) Comparison of the mean values of the top 5 stromal features between the Composite Adverse Outcome group and the No Outcome group. (b) Changes in the top 5 stromal features over transplant time. The blue lines indicate linear fit from the No Outcome group, and orange lines indicates the linear fit from the Composite Adverse Outcome group. Note that in the 4^th^ panel, there is an obvious and significant difference in the slopes for the two linear fits – a result that is obscured in the 4^th^ panel of this Figures section (a) which utilized average feature values instead of change over time values. (c) Illustrative examples of tissue from a patient in the Composite Adverse Outcome group and the No Outcome group. The first column corresponds to the original H&E image, in which the colored squares indicate the sampling sites: blue corresponds to endocardial stroma, and green corresponds to interstitial stroma. The second and fourth columns show the identified endocardial and interstitial stromal tissue after segmentation, respectively. The third and fifth columns show the discrete endocardial and interstitial fibers, with each fiber assigned a vector arrows. The number, length, and orientation of vectors indicate the density, length, and orientation of the segmented stromal fiber.

First, a U-net deep learning architecture was trained to segment cardiomyocytes. The U-net model with an encoding and a decoding component was executed in PyTorch framework on a Titan XGPU running CUDA 7.5 using previously described parameters.^28^ After obtaining the U-net model’s binary masks of the cardiomyocytes, the stroma and the blank areas were morphologically processed by disc-dilation, disc-corrosion, and disc-dilation operations. The binary masks after image morphology operations were then subtracted from the original masks to complete the partitioning of the stromal compartment. Figure 2b illustrates the effect of stroma being divided into three types. A local difference-local binary pattern operator^29^ combined with the Otsu algorithm was employed to detect and segment stromal fibers - an approach with established success for collagen fiber detection in other tissues.^30,31^ The success of automated stromal fiber segmentation was confirmed by experienced pathologists during pipeline development. An illustration of the stromal fiber segmentation is depicted in Fig. 3c. Source code for the digital pathology image analysis pipeline is freely available on Github.

### Data Analysis and Statistical Methods

To identify morphologic biomarkers of stromal remodeling, we compared the time-dependent response of each extracted stromal feature between the composite Adverse Outcome group (allograft loss or CAV grade >2 at 7-years) and the No Adverse Outcome group. Specifically, we constructed a mixed-effect regression model which includes variables for group, time, and the interaction term between group and time.^32^ In this model, the dependent variable was the examined stromal feature, and the independent variables were group and transplant time, with the interaction term formed by group and transplant time considered as a covariate to examine how stromal features respond to transplant time between different groups. By fitting this model, we were able to assess for significant differences in the stromal feature changes over time-from-transplant between the Adverse Outcome group and the No Adverse Outcome group. The five features that exhibited the most significant changes over time were selected and defined as the top features of ‘pathologic stromal remodeling’. Using these features of pathologic stromal remodeling, we examined the inflammatory history subgroups, comparing stromal feature change-over-time in EMBs from patients in each subgroup vs. Controls. Lastly, inflammatory history subgroups were individually assessed for event rates using the composite Adverse Outcomes data. All statistical analysis was conducted in python math package and Stata v.15.0 (StataCorp LLC). Figure 1 summarizes the experimental workflow from image analysis to data analysis.

## Results

### Cohort Summary

Summary clinical data for the study cohort is shown in Table 1. Overall, 71/650 patients (10.9%) experienced the composite Adverse Outcome. As expected, there were numerous clinical risk factors associated with the adverse outcomes of allograft loss or CAV grade ≥ 2 at 7-years, though Pearson correlation coefficients were generally modest (Table 3). The correlation coefficients of six clinical risk factors with composite outcomes exceeded 0.1, revealing a weak correlation. These six risk factors are re-transplantation, BMI at 1-year, Diabetes, CKD, Donor age, and Donor hypertension.

### Prioritizing the morphologic biomarkers of stromal remodeling

The top-5 stromal morphologic features associated with the composite Adverse Outcomes are listed in Fig. 3. Briefly, these stromal features describe the fiber density, solidity, and fiber orientation of the endocardial stroma, fiber thickness of interstitial stroma, and the area-ratio of cardiomyocytes to interstitial stroma. These five features each exhibit statistically significant differences (p < 0.05 for all) in their trends-over-time from transplant between the No Outcome group and the Adverse Outcome group using mixed-effects regression analysis (see Fig. 3b). A more simplistic approach to analyzing and interpreting stromal change based on per-patient averaging features measurements from each EMB a patient contributes to the cohort is shown in Fig. 3a. Notably, though some significant differences between the No Outcome and Adverse Outcome group persist in these averaging-based analyses, much of the nuance is lost with this approach (especially for stromal fiber thickness measurements). The feature measurement-over-time slopes presented in Fig. 3b better capture the progressive nature of stromal changes than the averaged feature measurements presented in the bar graphs in Fig. 3a, and represents a more statistically powerful and biologically meaningful method for studying quantitative morphologic data.

Figure 3c provides an intuitive visualization of the differences in stromal features. Examination of non-interstitial stroma in the second and third columns shows that the endocardial stroma from the No Outcome group is more compact, with fibers which are shorter, less thick, less parallel, and with more delicate crosslinks. Examination of interstitial stroma in the fourth and fifth columns shows that the area, density and length of interstitial stromal fibers are significantly increased in the Adverse Outcome group, suggesting proliferative interstitial remodeling.

### Exploring the effects of previous inflammatory events on stromal remodeling

**Figure 4:**
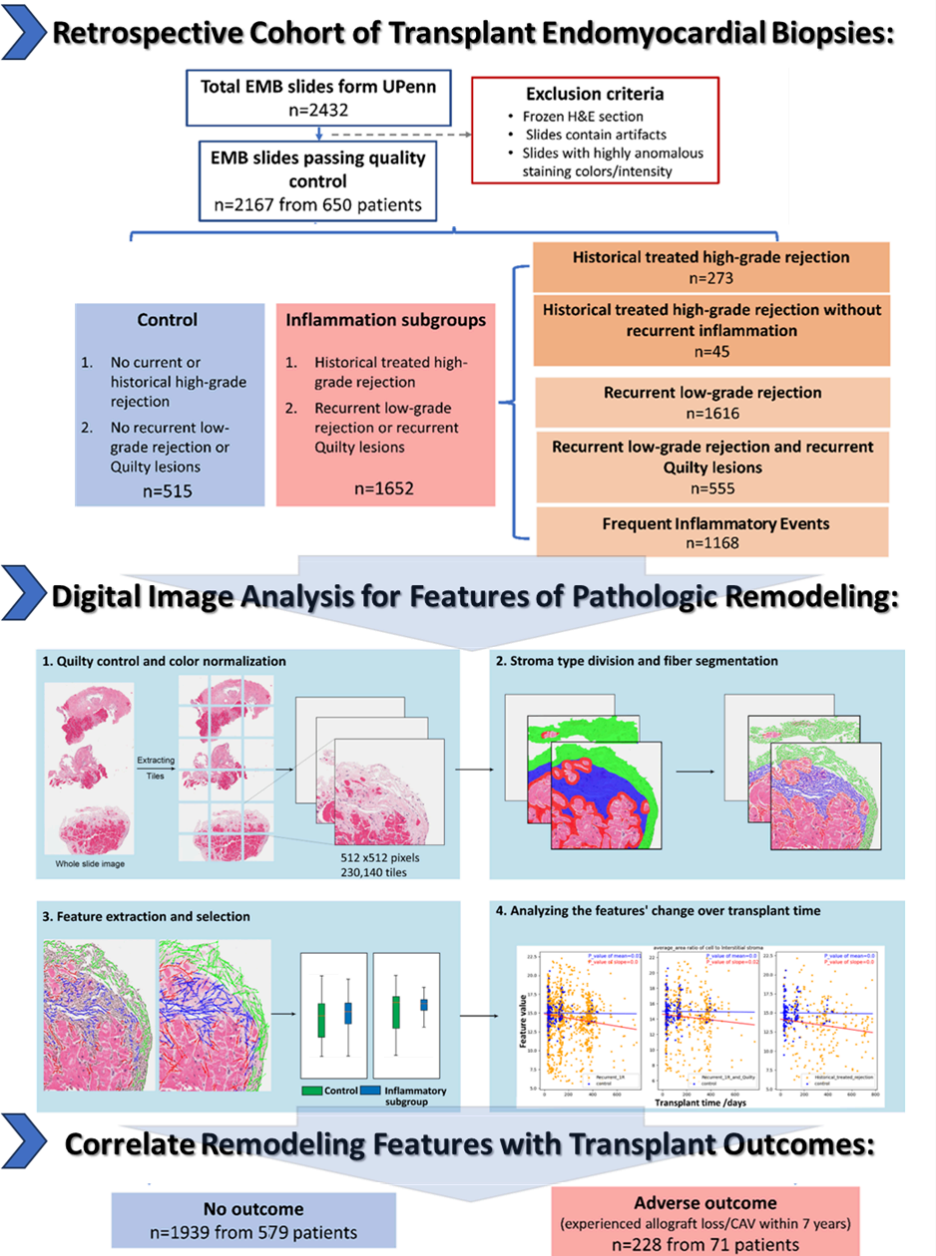
Time-dependent trajectories of prioritized morphologic biomarkers. Each row represents comparison to controls of one of the five patient subgroups based on a history of rejection diagnoses. Each column represents one of the morphologic biomarkers of pathologic rejection, selected based on strength of association with adverse patient outcomes. Blue linear fit lines represent the Control group (which is derived from the individual blue data points), while the Red linear fit lines represent the Inflammatory group (which are derived from the individual yellow data points). P-values generated via mixed effects modeling in each panel are derived from comparisons of linear fit slopes for Controls and Inflammatory groups.

Plots of the change-over-time for each morphologic biomarker of pathologic remodeling in each historical inflammation subgroup vs. controls are illustrated in Fig. 4. There are numerous statistically significant differences in the slopes of the feature-change-over-time plots, suggesting that alloimmune events detected on prior biopsies induce measurable stromal changes which can be detected on subsequent biopsies. Notably, significant differences in pathologic remodeling were observed not only as a result of long-recognized risk-factors like a history of high-grade/treated rejection, but also in the indolent inflammation subgroups which have experienced recurrent 1R events and/or recurrent Quilty. Additionally, when EMBs which also have a history of recurrent, indolent inflammation (eg. Recurrent 1R or Quilty) are excluded, a history of high-grade/treated rejection alone no longer manifests significant differences in most pathologic stromal remodeling features compared to Controls.

Examination of composite outcomes among patients contributing EMBs lends additional context to the in-situ stromal biomarker data. As seen in Table 4, patients contributing EMBs to several of the historical inflammation subgroups have significantly higher rates of adverse outcomes compared to patients contributing Control biopsies (5.1% event rate). These include patients contributing historical high-grade/treated rejection biopsies (32.9%, p< .001), patients contributing recurrent low-grade and recurrent Quilty biopsies (12.7%, p = 0.047), and patients contributing recurrent inflammation biopsies (19.5%, p < 0.001). The recurrent low-grade alone group demonstrated an event rate nearly double control patients (9.4% vs. 5.1%), though this was not significant (p = 0.16). Once again, a history of high-grade/treated rejection without a concurrent history of recurrent indolent inflammation did not confer increased risk (7.1%, p = 0.67). Taken together, the EMB and patient subgroups analyses suggest that inflammatory insults such as recurrent Quilty lesions and low-level rejection – which are typically not treated in clinical practice – induce measurable changes to the allograft stroma and may be associated with poor patient outcomes.

## Discussion

In this manuscript, we describe the development of a computational pathology analysis pipeline designed to comprehensively characterize the stromal architecture of cardiac allografts. Evaluating this pipeline on a large cohort of heart transplant EMBs, we examined the effects of allo-immunity from a novel perspective; focusing on the chronic stromal changes induced by inflammatory insults rather than on the inflammatory cells that induce those changes. Traditional histologic assessments of transplant EMBs such as ISHLT rejection grading focus predominantly on infiltrating inflammatory cells and their immediate effects.^1^ Recent computational pathology research in transplant medicine has focused predominantly on reproducing these traditional histologic assessments,^5,22^ and as a result, are largely constrained to the same, well-documented, limitations as the ISHLT grading framework. Our approach highlights the value of moving beyond this conventional framework, leveraging digital image analysis to monitor subtle morphologic changes occurring in EMBs over time, then identifying the core set of morphologic changes which portend poor long-term patient outcomes. We assert that future applications of digital pathology would benefit from adopting a similar approach, utilizing longitudinal samples and statistics to correlate progressive morphologic changes with hard clinical endpoints.

From a histopathology perspective, the morphologic features of pathologic remodeling we identified provide a detailed view of how the cardiac stroma is changed by different inflammatory insults. Historical inflammatory insults resulted in an increase in interstitial stroma area relative to myocyte area, a finding consistent with the myocyte loss and interstitial fibrosis that can result from immune-mediated myocardial injury.^5,11,15,18,20^ In addition, relative to controls, historical inflammatory insults increased stromal fiber thickness, solidity, and parallelism (eg. less disordered/branched fibers). This may be explained by progressive deposition of type I collagen after an inflammatory injury. Type I and type III collagen are the main structural constituents of the cardiac ECM, with type I collagen manifesting thicker, straighter, and more parallel fibers while type III collagen manifests finer, wavier, and more intricately branched fibers.^10,17,18^ It has been shown that rejection and other inflammatory insults can cause the proportion of type I collagen to increase relative to the type III collagen,^11,15,17,18^ resulting in a ‘stiffer’ myocardium. This may explain both the aforementioned stromal features which differentiated EMBs with more/more severe historical inflammatory events from Controls, and may explain the poorer long-term outcomes associated with these features. Lastly, although stroma area was increased, the apparent number/density of fibers in the stroma was reduced in EMBs with historical inflammation. Whether this results from different collagen subtypes, from non-fibrous ECM proliferation, from edema due to indolent inflammation, or from increased stroma ‘cellularity’ (which contributes to stroma area while increasing the space between individual fibers), cannot be definitively answered from this study. However, each is a potential mechanism worthy of exploration in future research.

The experiments reported in this manuscript yielded several findings of translational value. First, identifying the progressive stromal changes which are most strongly correlated with future adverse outcomes creates opportunities for intervention, either through augmented immunosuppression, through the use of traditional heart failure therapeutics with ‘reverse remodeling’ capability, or by application of new treatments which directly target stromal remodeling.^21^ Whether the specific biomarkers of pathologic remodeling uncovered in this experiment can be used to monitor treatment effects *after* a therapeutic intervention remains unknown, but is an additional potential application for the novel stromal biomarkers reported in this paper. Moreover, the finding that recurrent low-grade inflammatory processes are linked to adverse long-term outcomes is significant and worthy of further discussion.

It is common practice for transplant clinicians to monitor, but not treat, low-grade ACR events and Quilty lesions, only implementing acute or chronic therapeutic interventions for cases involving clinical evidence of allograft dysfunction. In fact, current ISHLT guidelines generally discourage treatment of low-grade ACR events.^33^ On the other hand, most episodes of ‘high-grade’ rejection as defined in this manuscript (either ACR ≥ 2R or pAMR > 0) undergo either acute treatment or alterations of chronic immunosuppression, largely in accordance with existing guidelines.^33,34^ In the present study, our results show that recurrent, indolent inflammatory processes like low-grade ACR and Quilty lesions are associated with significant, pathologic changes in the cardiac stroma, and that this leads to a higher incidence of adverse allograft outcomes. In addition, our results showed that isolated episodes of high-grade rejection in patients *without* a history of recurrent 1R or Quilty do not appear to induce significant long-term pathologic changes in the cardiac stroma. In the context of current practice patterns and guidelines, these findings suggest that clinicians may be under-valuing the importance of chronic ‘mild’ allo-immune responses, and may be – in some cases – over-valuing the impact of isolated, high-grade histologic rejection.

Prior research has shown correlations between Quilty lesions and adverse outcomes, though the available literature has conflicting findings.^1,2,20,35^ The impact of recurrent low-grade ACR on transplant outcomes has not been studied as frequently, though recent research does suggest that a history of higher ‘average’ rejection grades (even in the absence of high-grade events) is associated with a higher incidence of early CAV.^20^ While in this manuscript our findings generally support a connection between recurrent, untreated, indolent inflammation and adverse events, it is clear that not all patients with a history of Quilty and/or low-grade ACR necessarily suffer poor outcomes. Moreover, due the retrospective nature of this research, there is no way to assess the potential risks or benefits that might arise from altering immunosuppression based on a history of recurrent indolent inflammation. Nevertheless, given the strong correlation between pathologic remodeling features and poor outcomes in these patients, it is worth considering a potential clinical role for our stromal biomarkers. EMB samples are already obtained as part of routine care, and digital pathology analysis pipelines can be quickly and remotely accessed through cloud-based systems. Thus, while future clinical investigations are clearly needed, protocols which incorporate predictive morphologic biomarkers into immunosuppression management and CAV screening decisions might prove to be feasible and valuable.

As the field gradually pivots towards rejection surveillance paradigms which utilize more ‘liquid biopsy’ serologic assays and fewer EMBs,^6–9^ we assert that it will become increasingly important to rely on digital pathology biomarkers like those in this manuscript. If patients are to receive only 3–4 EMBs during their post-transplant course, then it is critical to extract maximum information from each of these events. The fewer EMBs performed, the lower the likelihood of identifying patients who are experiencing poor-outcome-associated recurrent 1R and Quilty lesions. Thus, it will be necessary to rely on surrogates for these recurrent histologic diagnoses, such as the biomarkers of pathologic stromal remodeling which we identified in this manuscript and which have clear associations with adverse outcomes. Future rejection surveillance protocols could therefore rely primarily on serologic testing, with EMBs performed at a few, widely spaced intervals to enable monitoring of subtle, serial changes which help identify at-risk populations. Compared to traditional, biopsy-heavy approaches relying on conventional histologic grading, such a hybrid approach could maximize personalization while still minimizing invasive testing.

As with all research, this study has limitations. Although the cohort comprised over 2000 biopsies, this was a single center study, and there were relatively few patients in the interesting ‘previous high-grade rejection without recurrent low-grade or Quilty’ subgroup. Additionally, while we utilized all available histologic diagnoses associated with study EMBs, additional, unmeasured, allo-immune processes could have confounded our findings. Due to limited application of immunostaining at our center on routine screening EMBs, our historical cohort precluded a complete and definitive assessment of AMR for in many cases. While we can confidently evaluate whether histologic criteria for AMR are met (i.e. pAMR(h+)), historical assessments of pAMR-(i+)) are limited to those EMBs which underwent clinical immunostaining at the time of EMB. Nevertheless, without pAMR(h+), without concurrent positive donor specific antibody testing, and without clinical evidence of rejection or provider decision to treat for rejection, we are confident that major rejection events were not mislabeled as a result of our center’s practice of intermittent/for-cause use of immune-staining on routine surveillance EMBs. Another unavoidable limitation of this study is reliance on pathology diagnostic records for assigning ‘inflammatory history’ case labels. It is well known that there is significant inter-pathologist variability in the application of ISHLT grades to transplant EMBs.^5,36,37^ Thus, study labels like ‘previous high-grade rejection’ or ‘recurrent low-grade rejection’ (without a history of high-grade rejection) are not definitively accurate. The fact that different pathologists would likely grade historical EMBs differently means that there is inevitable overlap between some study subgroups. It should be noted that while this limitation may affect subgroup comparisons, it has no impact on the correlations between specific patterns of stromal remodeling and patient-level clinical outcomes - a fact which further highlights the need for grounding computational pathology research in definitive clinical endpoints rather than imperfect histologic reference standards.

In conclusion, this study represents a novel and important application of computational pathology analysis within heart transplant medicine. Focusing on the allograft tissue itself rather than on the infiltrating immune cells, the stromal morphologic biomarkers described in this manuscript demonstrate the ability to quantify the effects of various historical inflammatory insults, uncovering new information about how different histories may predispose patients to adverse clinical outcomes.

## Figures and Tables

**Table 1:** Descriptive statistics for each of the inflammatory subgroups.

	Full Cohort 650)	Controls (n = 98)	Recurrent Low Grade Rejection (n = 519)	Recurrent Low Grade and Quilty (n = 166)	Historical Treated Acute Rejection (n = 85)	Historical Treated Rejection without Recurrent Low Grade/Quilty (n = 14)	> 5 Historical Inflammatory Events (n = 287)
**Recipient Race (%)**							
**• White**	71%	64.3%	73%	71.7%	62.4%	42.9%	63.8%
**• Black**	17.9%	18.4%	18.5%	21.1%	22.4%	35.7%	26.5%
**Hispanic/Latin**	2.1%	3.1%	1.9%	1.8%	3.5%	0%	3.8%
**• Asian**	1.4%	4.1%	0.8%	0.6%	1.2%	0%	0.7%
**• Not Specified**	7.6%	10.1%	5.8%	4.8%	10.5%	21.4%	5.2%
**Recipient Sex (% Male)**	76.7%	77.6%	77.8%	80.7%	72.9%	64.3%	77.7%
**Recipient Age at Transplant (average in yrs)**	52.3	53.9	52.2	50.3	48.1	43.7	51.7
**Re-Transplant**	3.8%	2%	3.9%	4.8%	8%	7.1%	3.1%
**BMI at 1 Year Post-Transplant**	28.2	30%	28.1%	28.3%	28.6%	27.4%	29%
**Recipient History of Hypertension**	32.9%	25.5%	33.5%	34.9%	50.6%	57.1%	45.6%
**Recipient History of Hyperlipidemia**	34.9%	25.5%	35.4%	36.1%	41.2%	35.7%	41.1%
**Recipient History of Diabetes**	37.7%	28.6%	38.7%	44.6%	36.4%	35.7%	40.4%
**Recipient Chronic Kidney Disease**	54.9%	53.1%	56.1%	46.4%	52.9%	42.9%	60.2%
**Recipient Race (%)**							
**HLA mismatch (average number** ^Ŧ^	4.4	4.5	4.4	4.6	4.6	4.6	4.6
**CPRA Score (% patients > 0)**	4.8%	8.2%	4%	3%	3.5%	0%	2.8%
**Positive Donor Specific Antibody** ^§^	7.1%	2%	7.5%	7.8%	16.5%	21.4%	14.3%
**Donor Age (average in years)**	37.2	37.3	37.1	36.2	39	32.1	37.4
**Donor Hypertension**	20.9%	21.4%	20.6%	16.3%	22.4%	21.4%	19.5%
**Donor Diabetes**	6.8%	5.1%	9.4%	7.2%	9.4%	7.1%	7.7%

Ŧ Defined based on number of mismatches in HLA-A, HLA-B, and HLA-DR, as per United Network for Organ Sharing database records.

§ Defined as the percentage of patients who contributed a biopsy to the cohort after having a diagnosis of de-novo donor specific antibody with mean fluorescent intensity > 1500

**Table 2:** The specific meaning, quantity, and source of the stromal biomarkers designed in this paper.

Feature family	Features extracted from	Specific features included	Statistics included in each feature	Quantity
**Stromal fiber Shape**	Endocardial stroma Interstitial stroma Scar-like stroma Overall stroma	Length: pixels in fiber orientation Thickness: average pixels in perpendicular to fiber orientation Size: total number of pixels Extent: ratio of pixels in the region to pixels in the total bounding box Eccentricity: ratio of the focal distance over the major axis length Solidity: ratio of pixels in the region to pixels of the convex hull image	Average Std Skewness Kurtosis Value range	4×7×5 = 140
**Stromal fiber orientation entropy**	Endocardial stroma Interstitial stroma Scar-like stroma	Local orientation entropy with 1) nearest 5 fibers and 2) nearest 10 fibers		3×2×5 = 30
**Stroma accumulation**	Endocardial stroma Interstitial stroma Scar-like stroma	Ratio of stromal region over whole slide Ratio of stromal region over cardiomyocyte		3X2×5 = 30
**Interaction between stromal fiber and myocardium**	Endocardial stroma Interstitial stroma	Angle between Interstitial fiber and cardiomyocyte surface, or between endocardial fiber and endocardial border.		2X1X5 = 10

**Table 3:** Correlation coefficients for clinical variables with composite adverse outcomes

Variable	Pearson Correlation Coefficient	Spearman Correlation Coefficient
**Recipient Sex (% Male)**	0.06	0.06
**Recipient Age at Transplant (average in yrs)**	−0.02	−0.02
**Re-Transplant**	0.16	0.17
**Body Mass Index at 1 Year Post-Transplant**	0.2	0.19
**Recipient History of Hypertension**	0.02	0.02
**Recipient History of Hyperlipidemia**	0.05	0.05
**Recipient History of Diabetes**	0.14	0.13
**Recipient Chronic Kidney Disease**	0.15	0.14
HLA mismatch (average number)*	0.02	0.02
**CPRA Score (% patients >0)** ^Ŧ^	−0.01	−0.02
**Positive Donor Specific Antibody** ^§^	−0.02	−0.02
**Donor Age (average in years)**	0.1	0.11
**Donor Hypertension**	0.15	0.15
**Donor Diabetes**	0.01	0.01

* HLA = Human leukocyte antigen. Mismatch defined by mismatch between donor and recipient as captured in the United Network for Organ Sharing records, which specifically captures mismatches at HLA loci A, B, and Dr.

Ŧ CPRA = calculated panel reactive antibody score, a measure of transplant recipient sensitization.

§ Positive donor specific antibody was defined as an antibody with mean fluorescent intensity > 1000.

**Table 4:** Adverse outcome event rate for patients contributing biopsies to each historical inflammation subgroup

Group	Number of patients contributing biopsies	Number of Events	Event Rate (P-value vs. control)
Control	98	5	5.1% (NA)
Recurrent Low-Grade Rejection	519	49	9.4% (0.16)
Recurrent Low-Grade & Quilty	166	21	12.7% (0.047)
High Grade/Treated Rejection	85	28	32.9% (<0.001)
High-Grade/Treated Rejection without Recurrent Low-Grade/Quilty	14	1	7.1% (0.67)
Recurrent Inflammation	287	56	19.5% (<0.001)

## Data Availability

The data that support the findings of this study are presented in the Manuscript and Extended Data sections. Unprocessed raw data is available from the corresponding author upon reasonable request.

## Abbreviations

EMB: endomyocardial biopsy
ISHLT: The International Society for Heart and Lung Transplantation
H&E: hematoxylin and eosin
CAV: early cardiac allograft vasculopathy
ACR: acute cellular rejection
AMR: Antibody mediated rejection
